# Mini Review: Molecular Interpretation of the IGF/IGF-1R Axis in Cancer Treatment and Stem Cells-Based Therapy in Regenerative Medicine

**DOI:** 10.3390/ijms231911781

**Published:** 2022-10-04

**Authors:** Syuan-Ling Lin, Chih-Yang Lin, Wei Lee, Chiao-Fang Teng, Woei-Cherng Shyu, Long-Bin Jeng

**Affiliations:** 1 Translational Medicine Research Center, China Medical University Hospital, Taichung 404327, Taiwan; 2 Translational Medicine Center, Shin-Kong Wu Ho-Su Memorial Hospital, Taipei 111045, Taiwan; 3 Graduate Institute of Biomedical Sciences, China Medical University, Yingcai Campus, Taichung 404333, Taiwan; 4 Organ Transplantation Center, China Medical University Hospital, Taichung 404327, Taiwan; 5 Department of Neurology, China Medical University Hospital, Taichung 404327, Taiwan; 6 Department of Occupational Therapy, Asia University, Taichung 41354, Taiwan; 7 Cell Therapy Center, China Medical University Hospital, Taichung 404327, Taiwan

**Keywords:** IGF, IGF-1R, monoclonal antibodies, cancer, stem cells, stem cell therapy, regenerative medicine

## Abstract

In addition to the fundamental role of insulin-like growth factor (IGF)/IGF-1 receptor (IGF-1R) signaling dysregulation in cancer initiation and proliferation, the IGF/IGF-1R signaling also plays an important role in the maintenance of stem cell characteristics and enhancement of stem cell-based therapeutic efficacy. This review focused on the role of IGF/IGF-1R signaling in preclinical IGF-targeted therapies, including IGF-1R monoclonal antibodies, IGF-1R tyrosine kinase inhibitors, and neutralizing antibodies of IGFs in multiple tumors and endocrine disorders. On the other hand, the function of IGF/IGF-1R signaling in stem cell self-renewal, pluripotency and therapeutic efficacy in regenerative medicine was outlined. Finally, the review summarized ongoing studies on IGF/IGF-1R signaling blockade in multiple cancers and highlighted the IGF-1R signaling modifications in stem cells as a potential strategy to improve stem cell-based therapeutics in regenerative medicine.

## 1. Introduction

Insulin-like growth factors (IGFs) play pivotal autocrine, paracrine and endocrine roles in the promotion of cell proliferation, differentiation and survival [1,2]. IGFs are members of a ligand family that includes insulin, IGF-1 as well as IGF-2, and they exert their action by binding to specific glycoprotein membrane receptors, namely, type 1 and type 2 IGF receptors (IGF-1R and IGF-2R), insulin receptors A and B (INSR-A and INSR-B) and hybrid receptors (IGF-1R/INSR-A and IGF-1R/INSR-B) [3,4]. Moreover, there is a noticeable homology among the IGF receptors, implying structural similarity and the possibility of signaling crosstalk [3]. Notably, IGFs binding to IGF receptors is regulated by soluble IGF binding proteins (IGFBPs), a family of six homologous molecules with high binding affinity for IGF-1 and IGF-2; IGFBP-3 is the most important of the homologous molecules, which binds to 80% of the IGF-1 [5]. IGF-1R has a tetrameric structure that is comprised of two alpha (α) subunits and two transmembrane beta (β) subunits linked by disulfide bonds [6]. The α subunits are the extracellular domain that directly binds to IGF, and each transmembrane β subunit contains an intracellular tyrosine kinase domain [7]. After engaging, the binding of IGFs induces a conformational change that activates the tyrosine kinase domain of the β subunit, leading to the autophosphorylation of specific tyrosine residues, which appears to be a critical step in receptor activation [8].

**Figure 1:**
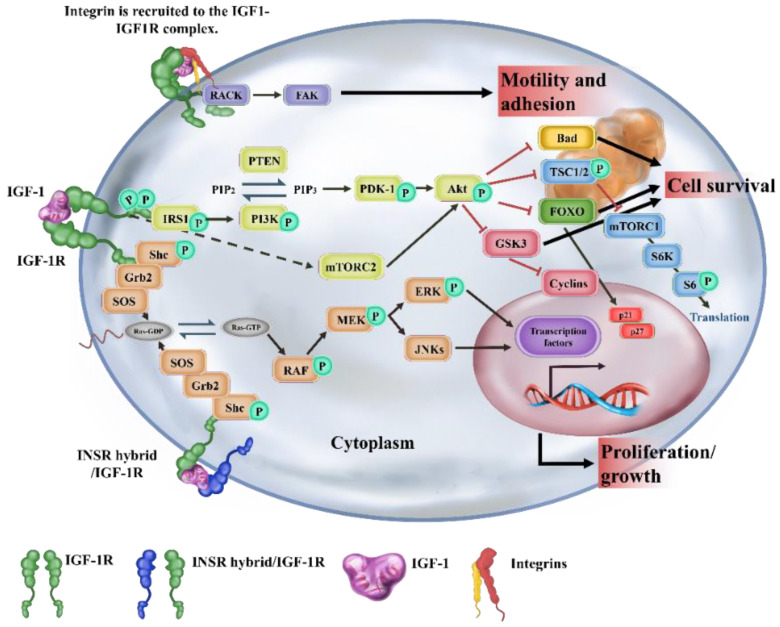
Downstream signaling of insulin-like growth factor-1 receptor (IGF-1R). Ligand binding to IGF-1R or IGF-1R/INSR hybrid receptors leads to the phosphorylation of tyrosines that create binding sites for docking proteins including IRS and Shc. Recruitment of IRS and Shc activates signaling via the PI3K/Akt and Ras/Raf/MAPK pathways, which regulate cellular proliferation, survival, migration, and metabolism. In addition to these pathways, interactions between IGF-1R and integrins, via scaffolding with RACK1 and FAK proteins, regulate cellular adhesion and motility. Black arrows indicate activation. Red arrows indicate inhibition. The symbol of P represents the site of phosphorylation. Akt, protein kinase B; ERK, extracellular-signal-regulated kinase; IRS, INSR substrate; MAPK, mitogen-activated protein kinase; MEK, mitogen-activated protein kinase/Erk kinase; mTORC1, mammalian target of rapamycin complex 1; PI3K, phosphatidylinositol-3-kinase; PTEN, phosphatase and tensin homolog; Shc, Src homology and collagen domain protein.

The IGF/IGF-1R signaling has been reported to play an important role in cancer development (Figure 1). Previous studies have reported that the activation of IGF/IGF-1R signaling is essential for cancer initiation and progression through several distinct pathways, including phosphorylation of mitogen-activated protein kinase (MAPK), which subsequently increases cell proliferation, the activation of phosphatidylinositol 3′ kinase (PI3K), which decreases apoptosis, and the regulation of mammalian target of rapamycin (mTOR) expression, which results in translational adaptation [9,10]. However, the activation of signaling pathways is also enhanced by other receptor tyrosine kinases (RTKs). For example, epidermal growth factor receptor (EGFR) enhances MAPK expression, promoting tumor cell proliferation and metastasis; fibroblast growth factor receptor (FGFR) increases PI3K/AKT expression, enhancing tumor angiogenesis; and hepatocyte growth factor receptor (HGFR or MET) enhances phosphorylated extracellular-signal-regulated kinase (ERK) expression, resulting in tumor survival and growth [11,12,13,14]. Notably, IGF-1R signaling has also been demonstrated to regulate the cancer stemness in various cancer stem cell (CSC) models, involving colorectal [15], breast [16], liver [17] and lung cancers [18]. IGF-1R signaling is also implicated in cancer development due to its stemness-related properties [16,19] and the resistance of radiation therapy and chemotherapy [20]. Furthermore, several studies showed that the elimination of cancer stemness by inhibiting IGF-1R signaling could be a considered way of targeting IGF/IGF-1R in cancer therapy [19,21]. However, several clinical trials of IGF/IGF-1R-targeting have experienced difficulty, leading to being terminated [22]. Hence, specific biomarkers for selecting suitable patients and the precise targeting of IGF-1R in CSCs are required. For example, the precise targeting of IGF-1R is feasible through the bioengineering of patient-derived chimeric antigen receptor (CAR)-T cells in sarcomas [23].

On the other hand, regenerative medicine is a major focus to determine novel therapies as well as to explore the biology and the pathogenesis of disease [24]. Recent advances in the isolation and development of stem cell have prompted scientists to identify and culture specific cell types for regeneration in various disorders including Parkinson [25], Alzheimer [26], or diseases of the heart [27], muscles [28], lung [29] and liver tissue [30]. Therefore, stem cells are considered efficient tools to treat tissue injuries in regenerative medicine. Several studies have indicated that growth factors including basic fibroblast growth factor (bFGF) and IGFs maintain the stemness and pluripotency of stem cells. Notably, the IGF/IGF-1R signaling has been reported to regulate the self-renewal and stemness of human embryonic stem cells (hESCs) [31,32] as well as the multipotent differentiation and repair capability of mesenchymal stem cells (MSCs). In hESCs differentiation, the overexpression of both IGF-1 and IGF-2 and IGF-1R phosphorylation lead to the differentiation of ESCs into hepatocytes [33,34]. In addition, the overexpression of IGF-1 in human bone marrow mesenchymal stem cells (BMSCs) has been reported to induce CXCL12/CXCR4 signaling in vitro. After transplantation in a rat model of permanent coronary artery occlusion, IGF-1 overexpression in BMSCs accelerated cell mobilization and retention in the injured area through the paracrine action of CXCL12/CXCR4 signaling to improve cardiomyocyte repair [35]. In this review, we discuss the clinical relevance of therapeutic strategies that target the IGF/IGF-1R axis in multiple malignant tumors and the applicability of IGF/IGF-1R axis strategies to improve stem cell-based therapies for human diseases.

## 2. Therapeutic Strategies Targeting IGF/IGF-1R Axis in Cancer

**Figure 2:**
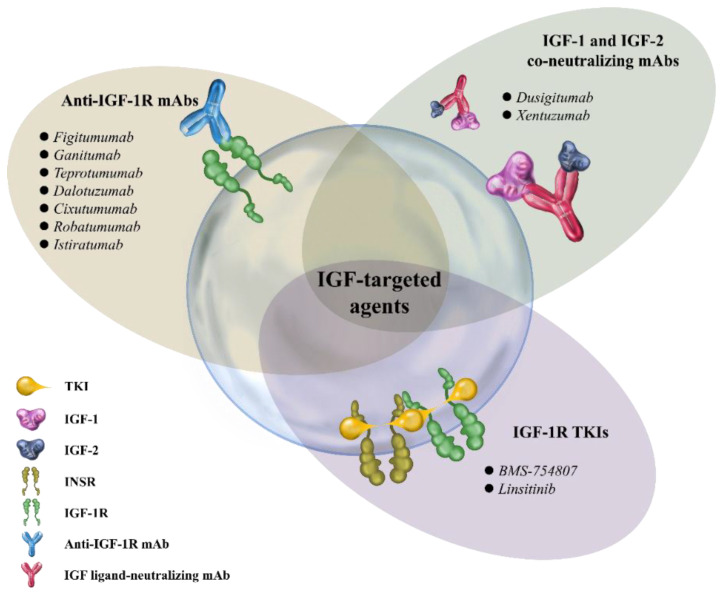
Examples of IGF-targeted agents. Anti-IGF-1R mAbs block ligand–receptor interactions and induce receptor internalization and degradation. Tyrosine kinase inhibitors bind to the receptor tyrosine kinase domain and block the downstream signaling of IGF-1R and INSR. IGF ligand-neutralizing mAbs bind to both IGF ligands (IGF-1 and IGF-2), thereby blocking the activation of IGF-1R and INSR-A. IGF-1R, insulin-like growth factor-1 receptor; mAbs, monoclonal antibodies; TKI, tyrosine kinase inhibitors; INSR, insulin receptor.

Recently, a number of different strategies targeting IGF/IGF-1R signaling have been developed for clinical evaluation, including anti-IGF-1R monoclonal antibodies (mAbs), small-molecule tyrosine kinase inhibitors (TKIs), and IGF-1 and 2 neutralizing antibodies (Figure 2). The following sections summarize the physical properties of each class of agents and their clinical evaluation status.

### 2.1. Anti-IGF-1R mAbs

Several studies have declared that IGF-1R is necessary for cellular oncogenes and is important in modulating the cell survival, motility, adhesion, and metastasis in multiple tumors and endocrine disorders [36,37]. For example, IGF-1R was frequently overexpressed in breast tumors, and its overexpression was important in the malignant transformation of mammary cells [38]. Moreover, the overexpression of IGF-1R provides breast tumors with an inherent resistance to radiotherapy and worsens the prognosis of patients [39]. These findings suggest that IGF-1R is a potential target for therapeutic interventions. The initial strategy involves the use of anti-IGF-1R mAbs to block ligand–receptor interactions and cause IGF-1R internalization and subsequent degradation [40,41]. Several therapeutic mAbs directed against IGF-1R have been developed, and encouraging preclinical outcomes have been observed with figitumumab (IgG2a, phase III) [42,43,44], ganitumab (IgG1, phase III) [45,46,47], dalotuzumab (IgG1, phase III) [48,49], teprotumumab (IgG1, phase II) [50,51], cixutumumab (IgG1, phase II) [52,53], robatumumab (IgG1, phase II) [54,55], and BIIB022 (IgG4, phase I) [56] in many malignant tumors. Moreover, istiratumab is a bispecific mAb that inhibits both IGF-1R and ErbB3 (IgG1 with two scFvs, phase II) [57] to kill malignant tumors. In addition, even though some IGF-1R mAbs for INSR exert negligible affinity, these IGF-1R mAbs still bind to their hybrid receptors, causing signal down-regulation. Some preclinical trials have reported Kirsten rat sarcoma virus (KRAS)-mutant tumors with high levels of free circulating IGF-1 in patients, and several reports have suggested that KRAS mutation has varying significance on the effect of IGF-1R inhibition, depending on the tumor type and/or molecular context. For instance, dalotuzumab has been used to treat KRAS wild-type colorectal cancer, with no evidence of anti-tumor activity [43]. However, this report indicated that the KRAS mutation enhanced IGF overexpression and resulted in PI3K activation, which were suppressed by targeting IGF-1R using figitumumab in non-small cell lung cancer cells (NSCLCs) [58]. Conversely, ganitumab was reported to be ineffective in sensitizing patients with KRAS-mutant colorectal cancer to folinic acid, fluorouracil and irinotecan (FOLFIRI) chemotherapy [59]. However, several clinical studies showed that the development of IGF-1R mAbs (figitumumab and ganitumab) was terminated, even though they exhibited preclinical activity against IGF-1R in some tumors, such as myeloma, prostate cancer, colorectal cancer and pancreatic cancer [22]. It is notable that the two mAbs, ganitumab and teprotumumab, which are used as monotherapeutic agents or in combination therapies are in ongoing clinical trials. For example, a combination chemotherapy with ganitumab in patients with Ewing sarcoma is currently in a randomized phase III trial (NCT02306161). Additionally, teprotumumab was evaluated in patients with moderate-to-severe thyroid-associated ophthalmopathy (TAO) in active stages, with a response in 69% of patients receiving the teprotumumab agent compared to a response in 20% of patients receiving a placebo agent in a randomized Phase II trial (NCT01868997) [60]. Subsequently, positive outcomes of the Phase III OPTIC trial of teprotumumab (NCT03298867) were reported in April 2019 [61]. Notably, teprotumumab was approved by the United States Food and Drug Administration (FDA) for use in TAO (2020) [62].

### 2.2. IGF-1R TKIs

An alternative approach to suppress IGF-1R signaling involves the treatment with small molecule IGF-1R TKIs that block IGF-1R kinase activity. As mentioned previously, approximately 85% of sequence homology was observed between the IGF-1R and INSR-A/B kinase domains, including the ATP-binding site [63]. Some studies indicated that ATP competitive antagonist of IGF-1R also inhibited the kinase activity of INSR [64,65]; therefore, linsitinib and BMS-754807 were frequently described as dual IGF-1R/INSR inhibitors [64,65]. For instance, linsitinib was reported to exert a superior anti-tumor activity of IGF-1R TKI and significantly blocked the compensatory INSR-A signaling. However, owing to the influence of linsitinib on metabolic insulin signaling through INSR-B, side effects including insulin resistance and hyperglycemia ensue [40,66]. Several studies have indicated the high expression of IGF signaling in many tumors and the sensitivity of tumors to IGF-1R inhibition. For example, the overexpression of IGF-1 in breast cancer cells in vitro was associated with poor prognosis of clinical cancers and correlated with the sensitivity to BMS-754807 in patients with triple-negative breast cancer in vivo [67,68]. Moreover, BMS-754807 has been known to have off-target effects in inhibiting the activation of tropomyosin-receptor-kinase A and B (TrkA and TrkB) and aurora kinases in pancreatic cancer [65], which influenced their therapeutic efficacy. However, some TKIs have exhibited promising preclinical activity in vitro; a few TKIs, including NVP-AEW541 [69], AZ12253801 [70] and BMS-536924 [71], have undergone clinical evaluation, and the results obtained have resulted in their termination. In addition, some TKIs belong to non-ATP competitive antagonists, which only inhibit IGF-1R; the INSR signaling is not affected, and these TKIs also suppress additional related genes. For instance, picropodophyllin (AXL1717) exhibits noticeable anti-tumor activity in multiple tumors through IGF-1R inhibition, leading to tumor regression [72,73], and AXL1717 also interferes with microtubule dynamics, which arrests G2/M phase [74]. Notably, the safety and efficacy of AXL1717 was demonstrated in a phase I clinical trial on patients with NSCLCs [75]. The other non-ATP competitive antagonist, nordihydroguai- aretic acid (masoprocol), was reported to inhibit cell proliferation of prostate tumor both in vitro and in vivo, but the clinical trial of nordihydroguaiaretic acid was terminated (NCT00678015) due to its hepatotoxicity and nephrotoxicity adverse effects [76].

### 2.3. IGF-Neutralizing Antibodies

This therapeutic approach involves neutralizing antibodies that directly target IGF-1 and IGF-2, thereby inhibiting survival signals through IGF-1R, INSR-A, and IGF-1R/INSR-A without interfering with INSR-B signaling and insulin action [77,78]. Therefore, these agents exhibit higher anti-tumor efficacy in multiple tumors and have a lower potential to cause hyperglycemia compared to IGF-1R TKIs [79,80,81]. Two IGF-neutralizing antibodies, including dusigitumab, a human IgG2λ monoclonal antibody with a high binding affinity for IGF-1 and IGF-2, inhibiting IGF-1R phosphorylation and INSR-A signaling, respectively, have been used in clinical trials [77]. Notably, dusigitumab suppresses the activation of IGF-1R and INSR-A, without binding to insulin, and thus retains insulin/INSR signaling [77]. Moreover, the phase I/II clinical trial of dusigitumab (NCT01446159) evaluated its anti-tumor efficacy and safety in combination with aromatase inhibitor in patients with metastatic breast cancer (hormone receptor (HR)-positive, and epidermal growth factor receptor 2-negative) [78]. The other antibody is xentuzumab, a IgG1 monoclonal antibody modified by humanization that binds to IGF-1 and IGF-2 with high affinity and blocks the IGF-1R and INSR-A functions [79]. Several studies showed that xentuzumab, in combination with other RTK inhibitors, was used in early clinical trials, including in EGFR-mutant NSCLC with afatinib (NCT02191891), in metastatic prostate cancer with enzalutamide (NCT02204072) and in HR-positive metastatic breast cancer with everolimus and exemestane (NCT02123823). Therefore, these combination agents demonstrated anti-tumor efficacy and a favorable safety profile, and they may serve as new promising therapeutic strategies in cancer therapy.

## 3. Activation of IGF/IGF-1R Signaling Improves Stem Cell-Based Therapeutic Strategies in Regenerative Medicine

Regenerative medicine is a new promising strategy to improve the treatment of tissue injury caused by trauma, disease, or aging. Owing to their self-renewal ability and pluripotency, stem cells are considered as efficient tools in preventing and treating diseases as well as tissue injuries. Moreover, several studies indicated that some factors and signaling pathways, such as bFGF [82], transforming growth factor β1 (TGF-β1) [83], leukemia inhibitory factor (LIF) [84], bone morphogenetic proteins (BMPs) [85], Wnt/β-catenin signaling [86], SMAD signaling [85], and MAPK signaling [87], maintain the stemness and pluripotency of stem cells. Notably, several studies suggest that IGF/IGF-1R axis activation is a possible regulator of the self-renewal and pluripotent capacities of stem cells through autocrine, paracrine, and receptor crosstalk. The following sections focus on IGF/IGF-1R signaling modification as a promising strategy to improve stem cell-based therapies for human diseases, including heart failure, neurodegenerative and neurological diseases, as well as bone disorders (Table 1).

### 3.1. Human Embryonic Stem Cells (hESCs)

Human ESCs are derived from the inner cell mass and contribute to multiple types of cells in the body [98]. Although many factors have been reported to maintain hESC populations, including bFGF [82,99], TGF-β1 [83,100], activin A [101], neurotrophins [102], Wnt/β-catenin signaling [86] and sphingosine-1-phosphate (S1P) [103], knowledge on the receptor activation required for the self-renewal of hESCs is limited. Notably, this study displayed the activation of IGF-1R signaling upon the co-culture of hESCs in the conditional medium of mouse embryonic fibroblast [31]. Furthermore, IGF-1R was overexpressed along with specific markers such as OCT4, SSEA4, and SSEA3. In addition, suppressing IGF-1R activation using IGF-1R mABs and knocking down IGF-1R expression by siRNA not only limits cell expansion but also promotes cell differentiation [31]. Moreover, the activation of IGF/IGF-1R signaling in hESCs promotes self-renewal by HRG/ERBB2 signaling and enhances cell survival through the PI3K/AKT pathway [31]. In addition, another study indicated that the activation of IGF-2/IGF-1R signaling is also essential for self-renewal, and high levels of IGF-2 maintain hESC proliferation and promote cell survival via a bFGF-dependent pathway [32]. Furthermore, IGF-1R signaling was implicated in the regulation of the pluripotency of hESCs. For example, this study indicated that IGF-1R signaling induced the proliferative ability of cardiomyocytes that differentiated from hESCs. Likewise, blocking IGF-1R activation using a IGF-1R mAbs decreased cardiomyocyte proliferation, while the addition of recombinant protein of IGF-1 or IGF-2 induced cardiomyocyte proliferation through the PI3K/AKT pathway [88]. However, this study demonstrated that microRNA-223 directly targeted IGF-1R and inhibited the AKT signaling, leading to hESCs differentiation [104]. The study also indicated that suppressing the activation of IGF-1R and its downstream signaling pathway induced hepatocyte differentiation from hESCs [34]. Therefore, these studies demonstrate that IGF-1R signaling is required for the maintenance of the stemness of hESCs, but the role of IGF-1R signaling in the differentiation of hESC needs to be elucidated.

### 3.2. Human Neural Stem Cells (hNSCs)

Human NSCs are derived from regions such as the subgranular zone (SGZ) in the hippocampal dentate gyrus and subventricular zone (SVZ) adjacent to the lateral ventricles. The hNSCs function as an additional tool for the development of regenerative therapy, and several clinical trials have indicated hNSCs as a promising target to treat neurological disorders, including amyotrophic lateral sclerosis (ALS) [105,106]. ALS is a lethal neurodegenerative disease that causes the rapid loss of motor neurons and muscular paralysis [107]. The report indicated that the overexpression of IGF-1 on hNSCs cultures enhances neural differentiation and promotes neuronal development by neurite outgrowth [95]. Furthermore, IGF-1-overexpressing hNSCs exhibit augmented neuroprotective effects against excitotoxicity, suggesting the potential of stem cell-based therapy in ALS [95]. In addition, another study indicated that hNSCs transplantation with IGF-1 transduction demonstrably improved injury-induced spatial learning deficits and decreased the activation of astroglial and microglial accumulation while enhancing the mobilization of oligodendrocyte precursor cells [108].

### 3.3. Cardiac Stem Cells (CSCs)

Although the reports on cardiac stem cells have been controversial in recent years, the IGF/IGF-1R axis has been reported to play a role in activating cardiac repair by governing CSCs survival, proliferation, migration and differentiation [109]. The IGF-1/IGF-1R activation is a basic cardioprotective mechanism associated with survival that improves ischemic cardiac function [96]. Moreover, a study indicated that c-kit+ expression in human CSCs promoted IGF-1 secretion, thereby enhancing IGF-1R signaling and ultimately improving cardiomyocyte survival [110]. IGF-1 overexpression in a subset of CD90+ CSCs was shown to activate IGF-1R signaling, promote stem cell survival, and protect surrounding cardiomyocytes from apoptosis after myocardial infarction. In intramyocardial transplantation, IGF-1-overexpressing MSCs are transplanted into the injured area, leading to preserved CSCs in the injured region; this enhances myocardial regeneration without differentiating into cardiac myocytes, smooth muscle, or endothelial cells [111]. Moreover, a study demonstrated that the mechanism of IGF-1 signaling activation promotes c-kit+ murine CSCs proliferation through the down-regulation of FOXO3/p27/p57 signaling [97]. Furthermore, the overexpression of IGF-1 promotes c-kit+/CD45- CSCs survival in obese mice, leading to significant improvements in cardiomyopathy caused by Western diet-induced obesity [112].

### 3.4. Mesenchymal Stem Cells (MSCs)

MSCs are multipotent adult stem cells with a great potential to differentiate into adipocytes, osteocytes, chondrocytes, neurons, and glial cells [113,114,115] and to maintain the self-renewal capability [116]. Several studies have demonstrated that IGF-1R signaling regulates MSC differentiation and maintains multipotency. For example, the addition of IGF-1 in adipocyte induction culture medium promotes adipogenesis in BMSCs by stimulating peroxisome proliferator-activated receptor-γ (PPAR-γ) expression and lipid accumulation [117]. A report also indicated that IGFs positively regulate the activation of AKT signaling, induce PPAR-γ expression and contribute to the maintenance of adipogenic differentiation in an AKT-1- and AKT-2-depleted animal model [118]. Moreover, the addition of bFGF enhances the autocrine IGF-1 and IGF-2 induction and induces IGF-1R activation, resulting in the adipogenesis and osteogenesis of UMSCs [89]. In addition to adipogenesis, IGF-1R signaling reportedly participates in osteogenic differentiation [90]. This study demonstrated that the autocrine IGFs/IGF-1R loop in BMSCs was activated and up-regulated through hedgehog signaling, which is required for osteoblast differentiation in skeletal development. Moreover, IGF-1R and its downstream AKT/mTOR signaling stabilize Gli2 protein and enhance the hedgehog-mediated effect on osteogenic differentiation [90]. Signaling via both IGF-1 and Runx2, which are osteogenic transcription factors, was up-regulated by serum response factor (SRF) to control bone remodeling. In mice with SRF-deleted osteoblasts, the transactivity of IGF-1 and Runx2 was restored through SRF overexpression, promoting osteoblastogenesis [119]. In addition, a few studies indicated that adding IGF-1 to hPMSCs under low-oxygen condition increased OCT4 expression, leading to cell proliferation and maintenance of the multipotency of hPMSCs through the activation of IGF-1R signaling [91,92]. In an animal model of acute myocardial infarction, the transplantation of adipose-derived MSCs (ADSCs) that overexpressed both IGF-1 and HGF to the injured area promoted neovascularization and suppressed inflammation. Although this report showed that both IGF-1 and HGF were overexpressed in ADSCs, significant cardiac regeneration was not observed [120]. Furthermore, several studies also demonstrated that IGF-1R activation in MSCs induces crosstalk and influences neighboring cells as well as their surrounding microenvironment, which could aid in tissue regeneration in human diseases. Stroke causes nervous system damage via ischemic and hypoxic changes to the injured brain, inducing muscle weakness and paralysis. Therefore, MSCs are considered as a promising therapeutic tool to treat stroke. For example, this study demonstrated that the intravenous injection of BMSCs in the brains of rats with stroke-induced middle cerebral artery occlusion increased IGF-1 expression and enhanced the activation of IGF-1/IGF-1R signaling, which corresponds to enhanced cell survival and neural progenitor cell recruitment to the injured area, leading to improved neurological functions [93]. Furthermore, the study reported that the transplantation of IGF-1R-overexpressing hDPSCs that crosstalk with CXCL12/CXCR4 signaling exhibited greater anti-apoptotic and anti-inflammatory effects as well as neural differentiation capacity in a cerebral ischemic animal model [94]. In addition, MSCs are consider to be a potential target in treating muscular injury and myocardial infarction. For example, IGF-1 addition to MSCs in vitro displayed the potential target for reducing scar formation, enhancing angiogenesis, promoting the reconstitution of muscle structure, and improving muscle function [121]. Furthermore, the in vivo transplantation of IGF-1-primed BMSCs represses cardiac dysfunction, increases the survival ability of engrafted BMSCs in the injured heart, leads to decreased cardiomyocytes cell apoptosis, and suppresses the expression of several inflammatory cytokines such as tumor necrosis factor-α (TNF-α), interleukin-1β (IL-1β), and IL-6 [122]. In addition, IGF-2 is an essential factor of the stem cell niche and has been reported to modulate the proliferation and differentiation of ADSCs. The study showed that the IGF-2/IGF-1R axis significantly induced the cell proliferation of ADSCs and increased the expression of stemness markers, such as Nanog, Oct4 and Sox2. Notably, IGF-2 had been reported to have a pivotal role in the differentiation of ADSCs to adipocytes and osteoblasts [123].

## 4. Conclusions

Studies on the role of IGFs in cancer have been conducted mainly on solid tumors including colon [124], prostate [124,125] and breast cancers [126]. For instance, high levels of IGF-1R in patients with colon cancer, as compared to healthy control, could indicate poor prognosis [127]. Therefore, a deeper understanding of cancer biology has led to the development of anti-tumor drugs targeting the specific oncogenic substrate IGF-1R. Since the IGFs/IGF-1R signaling is often dyseregulated in cancer development, it has been considered an attractive pharmacological target for solid tumors. This review summarizes the contribution of IGFs/IGF-1R signaling in the treatment of cancer and stem cells and also highlights the relevance of IGFs/IGF-1R signaling in potential strategies for cancer or stem cell-based therapies, respectively. In cancer, the dysregulation of IGFs/IGF-1R signaling can induce several hallmark genes of cancer and contribute to malignant transformation, tumor progression and resistance to a number of anti-tumor therapies such as radiation therapy and chemotherapy. Hence, the suppression of IGF-1R signaling is considered to be a promising strategy to inhibit tumor growth and improve survival in multiple cancers. Currently, several strategies have been developed to inhibit IGFs/IGF-1R signaling in cancer therapy. For example, teprotumumab, introduced in 2020, was the first licensed anti-IGF-1R agent for TAO treatment [62]. However, most anti-tumor IGF-1R mAbs or TKIs have not yet displayed significant benefits in patients randomly recruited to phase II/III clinical trials, which is probably due to the paucity of reliable predictive biomarkers of IGF-1R inhibition. Therefore, studies that target IGFs/IGF-1R in multiple tumors with suitable molecular contexts, including IGF-1R suppression, are being conducted to efficiently treat triple-negative breast cancers rather than HR-positive metastatic breast cancers [128,129,130] as well as treat KRAS-mutant NSCLCs rather than wild-type lung cancers [58]. Moreover, because the activation of IGFs/IGF-1R signaling results in the bypass pathway, the combination of IGF-1R mAbs/TKIs and other RTKs as anti-tumor agents is considered to a more effective strategy in cancer therapy.

On the other hand, IGFs are among the earliest growth factors to be found in a developing embryo and putatively act as autocrine and paracrine factors on several developing cells such as stem cells. This review emphasizes that IGF-1/IGF-1R signaling is an important functional pathway to identify stem cells and MSCs with superior self-renewal capacities as well as to determine the pluripotency of these cells. Furthermore, regulating IGF-1/IGF-1R signaling is a promising strategy to maintain stemness and improve the efficacy of stem cell-based therapies in several disease conditions. Moreover, IGF-1R signaling is also implicated in cancer development due to its stemness-related properties and resistance of radiation therapy and chemotherapy. Therefore, the activation of IGF-1R signaling in stem cell-based therapies wherein the stem cells differentiate into tissue-specific cell types in vitro deserves attention. Additionally, it is also imperative to research the use of the in vivo transplantation of genetically modified IGF-1R stem cells or MSCs to minimize the adverse consequences of tissue regeneration therapy and improve the therapeutic efficacy of regeneration in human diseases.

## Figures and Tables

**Table 1:** Therapy efficacy in stem cells and MSCs that overexpress IGF-1R in regenerative medicine.

Overexpression of IGF/IGF-1R in Stem Cells	Function	Pathway	Disease	Ref.
hESCs	Promoting self-renewal and survival	Self-renewal through HRG/ERBB2, and anti-apoptosis through PI3K/AKT	-	[31,88]
UMSCs	Adipogenic differentiation	bFGF induces IGF and FGF receptor	-	[89]
BMSCs	Osteogenic differentiation	Hedgehog pathway	-	[90]
PMSCs	Increasing cell proliferation and maintaining multipotency	Induce OCT4 expression	-	[91,92]
BMSCs	Promoting cell survival and neural progenitor cell recruitment	-	Ischemic stroke	[93]
DPSCs	Promoting neuroplasticity	Crosstalk between IGF-1/IGF-1R and CXCL12/CXCR4 pathway	Ischemic stroke	[94]
hNSCs	Promoting neuroprotection	-	Amyotrophic lateral sclerosis (ALS)	[95]
CSCs	Promoting cardiomyocyte survival and myocardial regeneration	FOXO3/p27/p51 pathway	Myocardial infarction	[96,97]

hESCs, human embryonic stem cells; UMSCs, umbilical cord-derived MSCs; BMSCs, bone marrow-derived MSCs; PMSCs, placental mesenchymal stem cells; DPSCs, dental pulp-derived MSCs; hNSCs, human neural stem cells; CSCs, cardiac stem cells.
